# Essential Components of Melanoma Histopathological Reporting: The Surgical Oncologist's Perspective

**DOI:** 10.1155/2018/9838410

**Published:** 2018-05-02

**Authors:** Vinka Nurdjaja, Masato Yozu, Jon A. Mathy

**Affiliations:** ^1^University of Auckland School of Medicine, Auckland, New Zealand; ^2^Histopathology Department, Middlemore Hospital, Counties Manukau District Health Board, Auckland, New Zealand; ^3^Plastic Surgery Unit, Middlemore Hospital, Counties Manukau District Health Board, Auckland, New Zealand; ^4^New Zealand Melanoma Institute, Auckland, New Zealand

## Abstract

Histopathological reporting plays a critical role in guiding the surgical oncologist's management plan in treatment of primary cutaneous melanoma. The International Collaboration on Cancer Reporting (ICCR) espouses various components of structured histopathological reporting as “essential” or “recommended.” From a surgical oncologist's perspective, we discuss the clinical relevance of each essential component, as well as prognostic and treatment implications with regard to treatment planning.

New Zealand and Australia possess the highest incidence of melanoma in the world [1]. In patients with newly diagnosed early-stage primary cutaneous melanoma, surgery remains the mainstay of initial treatment and is therefore usually orchestrated by the surgical oncologist. The surgical oncologist's management plan depends on patient characteristics and histopathological features of the primary lesion following excisional biopsy. Therefore, the histopathological report plays a central role in guiding initial treatment, staging, and prognosis advice provided to melanoma patients.

Here, we summarize for the clinician the relevance of the essential components of melanoma reporting from the surgical oncologist's perspective, emphasizing how these components influence patient prognosis and guide typical surgical management such as resection margins and sentinel node biopsy.

The ICCR guidelines were established following evaluation of existing histopathological guidelines by the Royal College of Pathologists (United Kingdom) (RCPath), Royal College of Pathologists of Australasia (RCPA), and College of American Pathologists (CAP) [2]. Current “essential” (mandatory/standard) and “recommended” (nonmandatory/guideline) components of structured histopathological reporting for primary cutaneous melanoma are summarized in Table 1 [3]. These components are important in evaluating prognosis, treatment options, candidacy for clinical trials, and standardized outcomes assessment. The preanalytical elements and components of macroscopic histopathological assessment are outside the scope of this article and are not discussed.

Primary lesion Breslow thickness bears the most significant prognostic and surgical implications and represents a cornerstone of American Joint Committee on Cancer (AJCC) staging [4]. While substaging is further determined by lesion ulceration, Breslow thickness represents the fundamental determinant of T-staging [4]. Furthermore, Breslow thickness acts as the basis of wide local excision (WLE) margins [5]. Current guidelines recommend invasive melanomas < 2 mm in thickness to be excised with at least 1 cm margin, while melanomas ≥ 2.0 mm are excised with at least 2 cm margins [5].

Breslow thickness is also an independent predictor of sentinel lymph node (SLN) status [6]. In a retrospective review of 221 patients undergoing sentinel node biopsy, there is SLN positive rate of 4.8% in T1 patients, 11.2% in T2 patients, 28.1% in T3 patients, and 46.5% in T4 patients [6]. Further data supports performing sentinel node biopsy (SNB) for microscopic staging of the regional lymph node basin when tumour thickness meets or exceeds 1 mm, or greater than or equal to 0.75 mm with other high risk features such as ulceration and/or high mitotic activity [7].

After Breslow thickness, both mitotic rate and ulceration reporting are important for the surgical oncologist in treatment planning. Mitotic rate is no longer considered a staging criterion for T1 melanoma in the most recent AJCC guidelines [8]. However, both features correlate with potential for metastatic spread and prognosis [2, 8]. Mitotic rate, as a sign of biologic activity, is particularly important in thin melanomas, where mitotic activity ≥ 1/mm^2^ is associated with a decrease in 10-year survival from 95% to 88% [9]. 5-year survival rate in ulcerated melanomas is proportionately worse than nonulcerated melanomas of the same T stage, while being similar to nonulcerated melanomas of the next highest T stage [9].

In addition to Breslow thickness, ulceration and mitotic rate can also influence the surgical decision to pathologically stage the regional lymph node basin with SNB. While SNB positivity has been reported at approximately 5% of all primary melanomas < 1 mm overall, the rate increases up to 20% for patients with thickness between 0.75 and 0.99 mm in the presence of mitotic rate ≥1/mm^2^ and/or ulceration [7]. This is important as melanomas ≤ 1 mm are reported to comprise over 70% of diagnoses made and also comprise 25% of all melanoma-specific deaths [10, 11].

The decision to perform SNB is important for the surgical oncologist, as the presence of regional lymph node metastasis represents the most significant prognostic factor in early-stage melanoma [7]. For T1 primary lesions, the 5-year Cancer Specific Survival (CSS) has been shown to fall from 94% to 69% when comparing SN-negative and SN-positive patients, respectively [11]. While results from the MSLT-II trial cannot be freely extrapolated to patients with heavy tumour or nodal burden, findings suggest that completion lymph node dissection should not be conducted in all patients with positive SN [12]. However, in appropriately selective patients, sentinel lymphadenopathy has also been shown to provide improved treatment outcomes in the node-positive cohort in terms of melanoma-specific survival, regional disease control, and surgical morbidity [13, 14]. A positive sentinel node biopsy also allows for an earlier opportunity to make informed decisions regarding candidacy for further investigations, treatments, and/or clinical trials and has implications for posttreatment surveillance [7]. There are numerous recent and pending clinical trials studying the effect of immunotherapy for early-stage metastatic melanoma, where studies have shown significant improvement in survival [15].

Margins of excision represent perhaps the most self-explanatory essential parameter of histological reporting. Widely clear margins are required to ensure clearance and to reduce local recurrence rate. Local recurrence is hypothesized to arise secondary to unresected microsatellites, intralymphatic spread, and/or intrinsic regional tumour influence [16]. Wide excisional margins performed per standard of care have been shown to reduce that risk [16]. For example, in T2 melanoma, local recurrence has been shown to fall from 3.6% to 0.9% after excision with 1 cm versus 2 cm margins, respectively [17]. Conversely, no significant difference in overall or recurrence-free survival has been shown between 1-2 cm margins and more radical 3–5 cm clinical margins [18].

Currently, there is little evidence to guide recommendations on depth of resection, as long as the deep resection margin is clear [18]. The ANZ 2008 guidelines recommend resection down to (not including) deep fascia [19], as supported by its physiologic tendency towards lymphatic blockade. Notably, another study has shown no difference in survival rates between patients with intact and resected deep fascia [16].

Satellites, defined as disconnected malignant cells greater than 0.05 mm in diameter divided by dermis at least 0.3 mm away from the primary invasive lesion, are postulated to reflect early metastatic activity along a spectrum with in-transit and regional lymph node metastases [9]. Both prognosis and SLN status are significantly affected, with significant reduction in survival and change in SLN positivity from 11 to 43% when comparing microsatellite with nonmicrosatellite groups [20]. Presence of ulceration with microsatellitosis reduces 5-year melanoma-specific survival from 83% to 43% [20].

Lymphovascular invasion (LVI) has also been shown to significantly correlate with prognosis and metastatic potential. Analysis of 2,243 patients with thin melanomas showed that LVI is an independent prognostic factor and is associated with increased SLN positivity [21]. Among patients with superficial spreading melanoma > 1.0 mm in thickness undergoing SLN biopsy, LVI was an independent risk factor reducing disease-free survival in the form of both local and in-transit recurrence [22]. A recent large analysis of all primary melanomas undergoing SLN biopsy has substantiated LVI as a predictor of SLN positivity independent of Breslow thickness [23], further reflecting the surgical oncologist's consideration of multiple tumour parameters in evaluating and managing nodal metastatic risk.

Desmoplastic melanoma (DM) represents a relatively rare variant of cutaneous melanoma with a clinical course more similar to soft-tissue sarcoma [24]. It is associated with increased local recurrence, possibly due to perineural skip lesions or missed areas of positive margin [24, 25]. Thus, surgical oncologists may steer towards wider local excision margins, as several studies show that this improves OS and reduces local recurrence [25]. Furthermore, there may be a role for adjuvant local radiation therapy in patients with DM to reduce risk of local recurrence [25]. Notably, further classifying DM into “pure” versus “mixed” subtypes may add clinical value, as mixed DM has been associated with poorer prognosis, including a worse 5-year melanoma-specific mortality of 31% compared to 11% in pure DM [25]. Conversely, pure DM may be associated with relatively improved prognosis compared to other melanoma subtypes, with similar mortality rates and lower rates of SLN positivity despite being 3 times thicker on average at time of diagnosis [25].

Perineural, intraneural invasion and “neural transformation,” in which the tumour forms neural structures, constitute neurotropism [2]. It is commonly found in DM but may also occur in other melanoma subtypes [2]. Neurotropic melanoma is associated with increased local recurrence and may compel the surgical oncologist to consider adjuvant radiotherapy and/or wider excisional margins [2].

Of note, Clark's level is no longer an essential component of structured reporting according to ICCR guidelines [2]. Clark's level has been largely superseded by mitotic rate as the more important parameter in characterizing melanomas after ulceration, and is no longer considered an independent prognosticator [9]. However, it still adds management value to Breslow thickness in extremely thin skin (e.g., eyelid skin or atrophic skin) and when mitotic rate cannot be accurately determined [9].

In summary, the essential elements of structured histopathological reporting contain invaluable information for the surgical oncologist. Although reporting has been shown to be most consistent for Breslow's thickness and Clark's level [1], each parameter has independent implications on prognosis and patient management. We hope that the clinical context provides a meaningful context to the role of the clinician in either reporting or interpreting of these elements in rendering individualised prognosis and treatment decisions for the patient.

## Figures and Tables

**Table 1:** Essential and recommended elements of structured microscopic histopathological reporting for primary cutaneous melanoma espoused by the International Collaboration on Cancer Reporting (ICCR) [3].

Breslow thickness	*Essential*
Surgical margin/tissue edge status	*Essential*
Ulceration	*Essential*
Mitotic count	*Essential*
Satellites	*Essential*
Lymphovascular invasion	*Essential*
Desmoplastic melanoma component	*Essential*
Neurotropism	*Essential*
Extent of ulceration	Recommended
Clark level	Recommended
Tumour-infiltrating lymphocytes	Recommended
Tumour regression	Recommended
Tumour regression margins	Recommended
Associated melanocytic lesion	Recommended
Intraepidermal melanoma growth pattern	Recommended
Melanoma subtype	Recommended
